# Prevalence of MSI‐H/dMMR Colorectal Cancer in Japan: Data From the Clinical Study Group of the University of Osaka‐Colorectal Registry

**DOI:** 10.1002/ags3.70226

**Published:** 2026-05-24

**Authors:** Yoshihiro Morimoto, Mamoru Uemura, Yoshinori Kagawa, Hidekazu Takahashi, Masayuki Hiraki, Yozo Suzuki, Shinichi Yoshioka, Mitsuyoshi Tei, Shu Okamura, Yukako Mokutani, Atsushi Naito, Shinji Tokuyama, Takamichi Komori, Akinobu Yasuyama, Genta Sawada, Yujiro Fujie, Shinya Yamashita, Naotsugu Haraguchi, Yusuke Matsuura, Chu Matsuda, Yoshinao Chinen, Tatsushi Shingai, Katsuki Danno, Nobuo Tanaka, Kazuya Sakata, Kohei Murata, Yuichiro Doki, Hidetoshi Eguchi

**Affiliations:** ^1^ Department of Gastroenterological Surgery Osaka General Medical Center Osaka Japan; ^2^ Clinical Study Group of the University of Osaka Osaka Japan; ^3^ Department of Gastroenterological Surgery Graduate School of Medicine, the University of Osaka Suita Japan; ^4^ Department of Gastroenterological Surgery Osaka International Cancer Institute Osaka Japan; ^5^ Department of Gastroenterological Surgery Osaka International Medical & Science Center, Osaka Keisatsu Hospital Osaka Japan; ^6^ Department of Surgery Kansai Rosai Hospital Amagasaki Japan; ^7^ Department of Surgery Toyonaka Municipal Hospital Toyonaka Japan; ^8^ Department of Surgery Yao Municipal Hospital Yao Japan; ^9^ Department of Surgery Osaka Rosai Hospital Osaka Japan; ^10^ Department of Surgery Suita Municipal Hospital Suita Japan; ^11^ Department of Gastroenterological Surgery Higashiosaka City Medical Center Higashiosaka Japan; ^12^ Department of Colorectal Surgery Sakai City Medical Center Sakai Japan; ^13^ Department of Surgery NHO Osaka National Hospital Osaka Japan; ^14^ Department of Gastroenterological Surgery Hyogo Prefectural Nishinomiya Hospital Nishinomiya Japan; ^15^ Department of Surgery Kaizuka City Hospital Kaizuka Japan; ^16^ Department of Surgery Itami City Hospital Itami Japan; ^17^ Department of Surgery Nishinomiya Municipal Central Hospital Nishinomiya Japan; ^18^ Department of Gastroenterological Surgery Nippon Life Hospital Osaka Japan; ^19^ Department of Gastroenterological Surgery Kindai University Nara Hospital Nara Japan; ^20^ Department of Gastroenterological Surgery Ikeda City Hospital Ikeda Japan; ^21^ Department of Surgery Japan Community Healthcare Organization Osaka Hospital Osaka Japan; ^22^ Department of Gastroenterological Surgery Otemae Hospital Osaka Japan; ^23^ Department of Surgery Kinki Central Hospital Itami Japan; ^24^ Department of Surgery Minoh City Hospital Minoh Japan; ^25^ Department of Surgery Kawachi General Hospital Higashiosaka Japan; ^26^ Osaka Prefectural Hospital Organization Department of Gastroenterological Surgery Osaka Habikino Medical Center Habikino Japan

**Keywords:** colorectal cancer, immune checkpoint inhibitor, japanese population, MSI‐H/dMMR, prevalence, surgery

## Abstract

**Objectives:**

Microsatellite instability‐high (MSI‐H) or mismatch repair‐deficient (dMMR) colorectal cancer (CRC) is a distinct subtype. Although immune checkpoint inhibitors are used for advanced cases, data on its frequency and characteristics, especially in resectable Asian CRC, remain limited. This study investigated MSI‐H/dMMR frequency and features in Japanese patients to support future personalized treatment strategies.

**Methods:**

This multicenter observational study included 1464 patients with pathologically confirmed primary CRC enrolled from April 2024 to March 2025 at 25 institutions affiliated with Osaka University. MSI/MMR status and clinicopathological data were extracted from a prospectively maintained database. Univariate analyses were performed to evaluate factors associated with MSI‐H/dMMR.

**Results:**

Among 1464 patients, 137 (9.4%) had MSI‐H/dMMR tumors. MSI/MMR testing was conducted preoperatively in 772 patients (52.7%). MSI‐H/dMMR was more frequent in patients aged ≥ 70 years, in females (OR = 1.46, *p* = 0.036), and in tumors located in the cecum, ascending, or transverse colon (*p* < 0.001). It was significantly less common in Stage IV disease (OR = 0.22, *p* < 0.001) and *KRAS*‐mutant tumors (OR = 0.22, *p* < 0.001), but strongly associated with *BRAF V600E* mutation (OR = 52.41, *p* < 0.001), poorly differentiated adenocarcinoma (OR = 7.17, *p* < 0.001), and mucinous adenocarcinoma (OR = 3.10, *p* = 0.002).

**Conclusions:**

MSI‐H/dMMR CRC accounted for 9.4% of Japanese patients, including resectable cases, and exhibited distinct clinical and molecular characteristics. These findings provide a basis for future personalized treatment strategies in this population.

## Graphical abstract

Using a multicenter Japanese colorectal cancer cohort from 25 institutions, we analyzed 1464 patients across all disease stages and tumor locations to determine the prevalence and clinicopathological features of MSI‐H/dMMR colorectal cancer. MSI‐H/dMMR tumors accounted for 9.4% of cases overall, were present in 22% of right‐sided colon cancers, and were observed in 11% of Stage 0–III disease. These findings characterize the distribution of MSI‐H/dMMR colorectal cancer in a large Japanese cohort and provide a basis for future personalized treatment strategies, including perioperative immunotherapy.

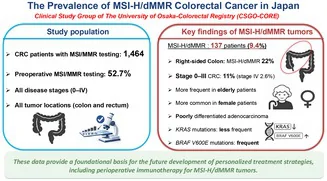

## 1 Introduction

Colorectal cancer (CRC) is one of the most common malignancies worldwide, including in Japan, with persistently high incidence and mortality rates [1, 2]. In recent years, subtype classification based on tumorigenic mechanisms and molecular alterations has advanced [3]. At the same time, the introduction of molecular targeted therapies and immunotherapy has highlighted the increasing importance of personalized medicine [4].

Among these subtypes, colorectal cancers with microsatellite instability‐high (MSI‐H) or deficient mismatch repair (dMMR) account for approximately 5%–15% of cases in large Western cohorts and are known to exhibit distinct clinical and pathological features compared to other molecular subtypes [5, 6, 7]. A previous large‐scale Japanese real‐world dataset of MSI testing performed as a companion diagnostic for pembrolizumab in unresectable or metastatic solid tumors reported an MSI‐H frequency of 3.78% among colorectal cancer samples, but it was not designed to provide colorectal cancer–specific analyses by anatomic subsite or stage, nor to evaluate co‐occurring molecular alterations [8]. In the present study, we included colorectal cancer cases across all stages and anatomical locations, with more than half of MSI/MMR testing performed preoperatively. Furthermore, we analyzed *KRAS* and *BRAF* mutation status and characterized the pattern of MMR protein loss, enabling a more comprehensive evaluation of MSI‐H/dMMR prevalence and its molecular context in colorectal cancer.

Recent studies have shown that immune checkpoint inhibitor (ICI) therapy is highly effective for MSI‐H/dMMR colorectal cancer. In Japan, ICIs are now reimbursed and have become the standard first‐line treatment for patients with unresectable or recurrent disease [4, 9, 10, 11, 12]. Perioperative ICI therapy is also gaining attention as a promising strategy for patients with resectable MSI‐H/dMMR colorectal cancer, with the potential to prevent recurrence and improve long‐term outcomes [13]. In Japan, preoperative testing for dMMR/pMMR status is not routinely performed in cases of resectable colorectal cancer, and the prevalence of MSI‐H/dMMR among these cases remains unclear. As treatment strategies for resectable MSI‐H/dMMR cases are expected to change in the near future, understanding their real‐world prevalence, clinicopathological characteristics, and associations with genetic alterations is critically important to facilitate timely clinical implementation.

This study aimed to clarify the prevalence and clinicopathological characteristics of MSI‐H/dMMR colorectal cancer, including resectable cases, in a Japanese population. The analysis will be based on prospectively registered cases of colorectal cancer over a one‐year period from April 2024 to March 2025 at Osaka University and 24 affiliated institutions. The findings from this study are expected to serve as fundamental data to support the future implementation of ICI therapy, including perioperative use, for colorectal cancer.

## 2 Methods

### 2.1 Study Population

This multicenter observational study targets colorectal cancer patients whose data were prospectively registered between April 1, 2024, and March 31, 2025, in a shared database established across all 25 affiliated institutions of Osaka University. Patients with pathologically confirmed colorectal adenocarcinoma, including all tumor stages and all tumor locations, were eligible for inclusion in this study. Patients with anal canal cancer or appendiceal cancer were excluded. No exclusion was applied based on the presence or absence of preoperative treatment, including neoadjuvant chemotherapy or chemoradiotherapy. During the study period, each participating institution adopted a policy to perform MSI testing or MMR protein analysis for all colorectal cancer cases whenever feasible, either preoperatively or postoperatively. Importantly, test eligibility was not selected or restricted based on patient or tumor characteristics. The present analysis included all cases in which MSI and/or MMR testing was actually performed.

### 2.2 Data Collection

The following clinicopathological variables were collected: age, sex, body mass index (BMI), medical history, tumor location, stage (UICC 8th edition), histological type, method of MMR/MSI testing, timing of MMR/MSI testing (preoperative or postoperative), pattern of MMR protein loss (MLH1, MSH2, MSH6, PMS2), MSI‐H/dMMR status, *KRAS/BRAF* mutation status, and type of surgical procedure.

### 2.3 MSI/MMR Testing

To evaluate the prevalence of MSI‐H/dMMR among Japanese patients, we assessed MSI/MMR status in consented colorectal cancer patients either preoperatively or postoperatively. MMR testing was performed at each participating institution using immunohistochemistry (IHC) with four antibodies: MLH1, MSH2, MSH6, and PMS2. MSI testing was outsourced and conducted using either PCR or next‐generation sequencing (NGS).

### 2.4 Ethical Considerations

This study was approved by the Ethics Committee of The University of Osaka (Approval No. 23323(T31)‐3). This was an observational study using information obtained during routine clinical practice. Information regarding the study's objectives, scope, target population, and data to be used was posted on the websites of the participating institutions. Informed consent was obtained using the opt‐out method, which allowed patients to decline participation. All collected data were anonymized and analyzed in a manner that did not allow individual identification.

### 2.5 Statistical Analysis

To evaluate associations between MSI‐H/dMMR status and clinical or molecular factors, univariate analyses were performed using 2 × 2 contingency tables. When all expected cell counts in the table were ≥ 5, odds ratios (ORs), 95% confidence intervals (CIs), and *p* values were calculated using univariate logistic regression, and statistical significance was evaluated using the Wald test. When any expected cell count was < 5, Fisher's exact test (two‐sided) was applied, and ORs were calculated accordingly. In such cases, 95% CIs were omitted due to limited sample size and the conservative nature of the test. The reference categories for OR calculation were clearly designated in the tables. In the analysis of associations between MLH1 deficiency and *RAS/BRAF* mutation status, all comparisons were performed using Fisher's exact test due to the presence of small or zero cell counts. All analyses were performed using JMP Version 17 (SAS Institute Inc., Cary, NC, USA). A two‐sided *p* value < 0.05 was considered statistically significant.

## 3 Results

### 3.1 Clinicopathological Characteristics of the Study Population

Table 1 summarizes the clinicopathological characteristics of the 1464 patients who underwent MSI/MMR testing. The median age was 73 years (range: 26–97), and the median body mass index (BMI) was 22.1 (range, 12.2–39.5). The cohort comprised 819 men (55.9%) and 645 women (44.0%). Regarding tumor location, 531 patients (36.2%) had tumors in the right‐sided colon (cecum, ascending, and transverse colon), 598 (40.8%) in the left‐sided colon (descending, sigmoid, and rectosigmoid colon), and 335 (22.9%) in the rectum. MSI‐H/dMMR was identified in 137 patients, accounting for 9.4% of the cohort. Notably, preoperative MMR or MSI testing was performed in 772 cases, representing 52.7% of the total cohort. Of the 137 MSI‐H/dMMR cases, 39 patients were diagnosed based on MSI testing alone as MSI‐H, while 98 patients were identified as dMMR by immunohistochemistry. Regarding MMR protein expression patterns, loss of MLH1 and PMS2 was the most frequent finding (*n* = 72). Other less frequent MMR protein deficiency patterns, including isolated and combined losses of MSH2, MSH6, and PMS2, were identified, as detailed in Table S1.

**TABLE 1:** Clinicopathological characteristics of patients (*n* = 1464).

Sex	
Male	819 (55.9%)
Female	645 (44.0%)
Age, median (range)	73 (26–97)
Body mass index, median (range)	22.1 (12.2–39.5)
Tumor location	
Right‐sided colon	531 (36.2%)
Left‐sided colon	598 (40.8%)
Rectum	335 (22.9%)
MSI‐H/dMMR	137 (9.4%)
Pre‐operative MSI/MMR testing	772 (52.7%)

*Note:* Left‐sided colon includes rectosigmoid colon.

Abbreviations: dMMR, deficient mismatch repair; MSI, microsatellite instability.

### 3.2 Clinical and Molecular Factors Associated With MSI‐H/dMMR

Table 2 summarizes the associations between clinical and molecular factors and the presence of MSI‐H/dMMR. MSI‐H/dMMR was significantly more common in older patients. Compared to those aged 60–69 years (referent), patients aged 70–79 years (OR = 1.90, *p* = 0.029) and ≥ 80 years (OR = 2.93, *p* < 0.001) had higher odds of being MSI‐H/dMMR. MSI‐H/dMMR was significantly more common in female patients than in male patients (OR = 1.46, *p* = 0.036). Tumor location was strongly associated with MSI‐H/dMMR status. Compared with the sigmoid colon, tumors in the cecum (OR = 10.46), ascending colon (OR = 14.86), and transverse colon (OR = 11.23) had significantly higher odds of being MSI‐H/dMMR (*p* < 0.001 for all). MSI‐H/dMMR was more common in early‐stage tumors. Compared with Stage II, Stage IV tumors showed significantly lower odds of being MSI‐H/dMMR (OR = 0.22, *p* < 0.001). With respect to histology, poorly differentiated adenocarcinoma (OR = 7.17, *p* < 0.001) and mucinous adenocarcinoma (OR = 3.10, *p* = 0.002) were significantly associated with MSI‐H/dMMR compared to moderately differentiated adenocarcinoma (referent). In contrast, well‐differentiated adenocarcinoma and papillary adenocarcinoma did not show significant associations with MSI‐H/dMMR compared with the referent group. Medullary carcinoma was observed in only one case in each group, and statistical significance could not be determined due to the limited sample size. In molecular analyses, tumors harboring *KRAS* mutations had significantly lower odds of being MSI‐H/dMMR compared to *RAS* wild‐type tumors (OR = 0.22, *p* < 0.001). In contrast, *BRAF V600E* mutations were strongly associated with MSI‐H/dMMR (OR = 52.41, *p* < 0.001).

**TABLE 2:** Association between clinical and molecular factors and MSI‐H/dMMR.

	MSS/MSI‐L/pMMR (*n*)	MSI‐H/dMMR (*n*)	dMMR (%)	OR of being MSI‐H/dMMR	95% CI	*p*
Age, years						
< 40	13	0	0.0	0.0	—	1.000*
40–49	76	1	1.3	0.22	—	0.138*
50–59	184	13	6.6	1.19	0.56 to 2.54	0.648
60–69	270	16	5.6	Referent		
70–79	479	54	10.1	1.90	1.07 to 3.39	**0.029**
≥ 80	305	53	14.8	2.93	1.64 to 5.25	**< 0.001**
Sex						
Female	573	72	11.2	1.46	1.02 to 2.07	**0.036**
Male	754	65	7.9	Referent		
BMI, kg/m^2^						
< 25	1016	112	9.9	Referent		
≥ 25	311	25	7.4	0.73	0.46 to 1.15	0.171
Location						
Cecum	98	23	19.0	10.46	4.36 to 25.12	**< 0.001**
Ascending	192	64	25.0	14.86	6.67 to 33.09	**< 0.001**
Transverse	123	31	20.1	11.23	4.82 to 26.19	**< 0.001**
Descending	69	3	4.2	1.94	—	0.401*
Sigmoid	312	7	2.2	Referent		
Rectosigmoid	203	3	1.5	0.66	—	0.747*
Rectum (Ra)	164	1	0.6	0.27	—	0.275*
Rectum (Rb)	166	5	2.9	1.34	—	0.760*
Stage						
0–I	156	25	13.8	1.33	0.80 to 2.21	0.268
II	465	56	10.7	Referent		
III	442	49	10.0	0.92	0.61 to 1.38	0.689
IV	264	7	2.6	0.22	0.10 to 0.49	**< 0.001**
Histology						
Well‐differentiated	477	38	7.4	0.94	0.62 to 1.43	0.780
Moderately differentiated	733	62	7.8	Referent		
Poorly differentiated	33	20	37.7	7.17	3.88 to 13.23	**< 0.001**
Papillary adenocarcinoma	38	4	9.5	1.24	—	0.565*
Signet‐ring cell carcinoma	3	1	25.0	3.94	—	0.280*
Mucinous adenocarcinoma	42	11	20.8	3.10	1.52 to 6.31	**0.002**
Medullary carcinoma	1	1	50.0	11.82	—	0.152*
*RAS* status						
*RAS* wild	637	93	12.7	Referent		
*KRAS* mutant	506	16	3.1	0.22	0.13 to 0.37	**< 0.001**
Other mutant	49	2	3.9	0.28	0.07 to 1.17	0.081
NA	135	26	16.1	1.32	0.82 to 2.12	0.251
*BRAF* status						
*BRAF* wild	1146	43	3.6	Referent		
*BRAF/V600E*	30	59	66.3	52.41	30.71 to 89.46	**< 0.001**
Other mutant	10	9	47.4	23.99	—	**< 0.001** *
NA	141	26	15.6	4.91	2.93 to 8.24	**< 0.001**

*Note:* Referent = reference category for odds ratio calculation. Stage classification based on UICC. BMI classification: < 25 = Nonobese, ≥ 25 = Obese.

Abbreviations: CI, confidence interval; dMMR, deficient mismatch repair; MSI‐H, microsatellite instability‐high; NA, not available; OR, odds ratio; Ra, rectum above the peritoneal reflection; Rb, rectum below the peritoneal reflection.

* Fisher's exact test. Bold values indicate statistically significant differences (*p* < 0.05).

### 3.3 Association Between MLH1 Deficiency and *RAS/BRAF* Mutation Status

Table 3 summarizes the association between deficient MMR protein patterns and *RAS/BRAF* mutation status among patients with dMMR colorectal cancer only. MLH1‐deficient tumors were significantly less likely to harbor *RAS* mutations compared with tumors showing preserved MLH1 expression (OR = 0.13, *p* = 0.009). In contrast, *BRAF* mutations were observed exclusively in MLH1‐deficient tumors (*p* < 0.001). In an additional analysis, we compared *RAS* mutation status between MLH1‐proficient dMMR tumors and MSS/MSI‐L/pMMR tumors (Table S2). Although the number of MLH1‐proficient dMMR cases was limited, the frequency of *KRAS* mutations did not differ significantly between the two groups.

**TABLE 3:** Association between MLH1 deficiency and *RAS/BRAF* status.

	MLH1 proficient (*n*)	MLH1 deficient (*n*)	OR	*p*
*RAS* status				
Wild	6	56	Referent	
Mutant	5	6	0.13	**0.009**
Unknown	10	15	0.16	**0.002**
*BRAF* status				
Wild	10	17	Referent	
Mutant	0	46	—	**< 0.001**
Unknown	11	14	0.75	0.778

*Note:* MLH1‐proficient were defined as cases with intact MLH1 expression and loss of one or more other mismatch repair proteins (MSH2, MSH6, and/or PMS2).

Abbreviation: OR, odds ratio. Bold values indicate statistically significant diferences (*p* < 0.05).

## 4 Discussion

In this study, we analyzed 1464 patients with colorectal cancer to investigate the association between MSI‐H/dMMR status and clinicopathological as well as molecular features in a Japanese population. MSI‐H/dMMR was observed in 9.4% of the entire cohort. A distinctive feature of this study is the inclusion of a substantial proportion (52.7%) of patients who underwent MMR or MSI testing preoperatively.

To date, large‐scale reports from Japan have primarily focused on the frequency of MSI‐H/dMMR in unresectable advanced and recurrent colorectal cancer, with data mostly derived from Stage IV cases or resected primary tumor specimens. Fujiyoshi et al. analyzed 2439 resected colorectal cancer patients across all disease stages and reported stage‐stratified MSI‐H prevalence, demonstrating a higher frequency in Stage II and a lower frequency in Stage IV disease, findings that are concordant with the present study [14]. However, the primary focus of their analysis was the detailed metastatic pattern of Stage IV colorectal cancer rather than a comprehensive evaluation across all disease stages and tumor locations. In contrast, the present study includes a considerable number of cases with preoperative MSI/MMR testing and comprehensively covers all disease stages and tumor locations, thereby providing a novel large‐scale evaluation of MSI‐H/dMMR prevalence in a Japanese clinical setting.

MSI‐H/dMMR was significantly more common among older patients, with the prevalence increasing in those aged 70 years and above, and the highest odds ratio observed in patients aged 80 years or older. This trend is considered to be associated with age‐related changes, particularly the progressive epigenetic silencing of the MLH1 gene due to promoter methylation as aging advances [15, 16].

Previous studies have suggested a bimodal age distribution of MSI‐H tumors, with early‐onset cases linked to Lynch syndrome and late‐onset cases associated with sporadic MLH1 promoter methylation [5]. However, such a clear bimodal pattern was not observed in our study, possibly due to the sample size or selection bias.

With respect to anatomical location, MSI‐H/dMMR was significantly more common in right‐sided colon cancers, whereas its prevalence was lower in left‐sided colon and rectal cancers. This trend is consistent with previous studies and may reflect embryological differences and the distinct molecular pathogenesis characteristic of right‐sided colorectal cancers [17, 18].

MSI‐H/dMMR was more commonly observed in early‐stage disease, whereas its frequency was markedly lower in Stage IV. This finding suggests that tumors with MSI‐H/dMMR may be less likely to develop distant metastasis, possibly due to increased immune recognition driven by a high neoantigen load [19, 20]. Histologically, poorly differentiated adenocarcinoma and mucinous adenocarcinoma were significantly associated with MSI‐H/dMMR, which is consistent with previous reports [21]. At the molecular level, MSI‐H/dMMR showed a significant negative association with *KRAS* mutations, whereas a strong positive association was observed with *BRAF V600E* mutations. *BRAF V600E* is a representative molecular alteration in sporadic MSI‐H colorectal cancers caused by MLH1 promoter methylation, and this trend was clearly confirmed in the present study [22, 23]. Furthermore, all cases with *BRAF* mutations in the dMMR subgroup exhibited MLH1 loss, supporting the previously reported high concordance between MLH1 promoter methylation and *BRAF* mutations in sporadic MSI‐H/tumors [22, 23, 24]. In contrast, *RAS* mutations were negatively associated with MLH1 loss and were mutually exclusive with *BRAF* mutations [25]. Accumulating evidence indicates that MSI‐H/dMMR tumors and *RAS*‐mutant colorectal cancers represent largely distinct molecular pathways in colorectal carcinogenesis [26, 27]. This concept is further supported by the Consensus Molecular Subtypes (CMS) classification, in which MSI‐H/dMMR tumors are predominantly classified as CMS1 (MSI‐immune), whereas *KRAS*‐mutant tumors are mainly enriched in CMS3 (metabolic), highlighting their biological divergence [28]. This molecular separation has also been observed in Japanese multi‐institutional cohorts. Large‐scale molecular profiling studies from Japan have consistently demonstrated that MSI‐H/dMMR tumors, often associated with MLH1 deficiency and *BRAF* mutations, show a molecular landscape distinct from *KRAS*‐driven MSS tumors, supporting the applicability of this framework to Japanese patients with colorectal cancer [29].

First‐line ICI therapy for MSI‐H/dMMR metastatic colorectal cancer has already become the global standard of care. In recent years, evidence has also emerged supporting the efficacy of perioperative ICI therapy for resectable MSI‐H/dMMR colorectal cancer. For colon cancer, the NICHE‐2 study reported that neoadjuvant ICI therapy with nivolumab and ipilimumab for resectable MSI‐H/dMMR colon cancer achieved a pathological complete response rate of 68% and a 3‐year recurrence‐free survival rate of 100% [30]. Furthermore, in the NEOPRISM‐CRC trial, 32 patients with Stage II–III colon or rectal cancer and a high or intermediate tumor mutational burden (≥ 6 mutations/Mb) received three courses of neoadjuvant pembrolizumab, achieving a pathological complete response in 59% of cases, with only 6% experiencing Grade 3/4 adverse events [31]. In the present study, we also demonstrated that MSI‐H/dMMR tumors account for more than 10% of resectable colorectal cancers in Japan. Clinical trials are currently underway in Japan as well, including the AZUR‐2 trial for colon cancer and the VOLTAGE‐2 trial for resectable rectal cancer, both investigating the utility of perioperative ICI therapy. These ongoing studies, together with further investigations into the safety and efficacy of such approaches, may significantly reshape the treatment strategy for resectable MSI‐H/dMMR colorectal cancer in the near future.

In addition, advanced rectal cancer with MSI‐H/dMMR can be regarded as a rare subgroup with a distinct biological profile from MSS/pMMR rectal cancer, and it requires careful consideration of specific challenges in treatment strategy. In clinical practice, these cases are more frequently seen in elderly female patients, in whom the omission of lateral lymph node dissection may be considered due to its physical burden. Moreover, oxaliplatin‐based adjuvant chemotherapy is often difficult to administer in such patients. Therefore, to achieve favorable treatment outcomes, it is necessary to establish individualized treatment approaches that differ from those used for MSS/pMMR cases.

The present study is particularly noteworthy because it is a large‐scale analysis in Japan to report the prevalence of MSI‐H/dMMR colorectal cancer across all disease stages, including a substantial proportion of preoperative cases. These results provide valuable real‐world insights and may serve as a cornerstone for the future clinical application of perioperative immune checkpoint inhibitor therapy. However, several methodological limitations of this study should be acknowledged. First, this study was not designed as a consecutive cohort enrolling all eligible patients, and therefore some degree of selection bias is unavoidable. Although participating institutions made efforts to harmonize MSI/MMR testing practices and to perform testing whenever feasible irrespective of patient characteristics, patients who underwent MSI/MMR testing were significantly younger than those who did not, suggesting a potential age‐related testing bias. In addition, early‐stage tumors (Stage 0–I) were overrepresented in the untested group, possibly reflecting insufficient tumor volume or limited clinical indication for molecular testing in these cases (Table S3). Consequently, the prevalence estimates reported herein may not fully reflect the true population‐level prevalence. Second, the present analyses were restricted to univariate comparisons. Future studies involving larger and more representative cohorts will enable multivariable logistic regression analyses to clarify independent associations with MSI‐H/dMMR colorectal cancer. Third, analyses examining the association between MMR protein deficiency patterns and *RAS/BRAF* mutation status were confined to patients with dMMR colorectal cancer, resulting in a limited number of MLH1‐proficient cases. This imbalance may have constrained statistical power and limited the generalizability of the observed associations. Finally, although MSI‐H/dMMR status and specific MMR protein loss patterns may suggest an underlying germline predisposition, germline mutation status was not systematically assessed in this study. Future large‐scale, prospective studies integrating comprehensive clinical and molecular evaluation will be required to further elucidate the contribution of hereditary factors to MSI‐H/dMMR colorectal cancer.

## 5 Conclusions

This study clarified the frequency and clinicopathological features of MSI‐H/dMMR colorectal cancer in the Japanese population, including resectable cases. The findings provide important foundational data for the development of new treatment strategies for MSI‐H/dMMR colorectal cancer.

## Author Contributions

**Yoshihiro Morimoto:** conceptualization, methodology, data curation, investigation, formal analysis, visualization, project administration, writing – original draft. **Mamoru Uemura:** conceptualization, methodology, writing – review and editing, validation, project administration, supervision. **Yoshinori Kagawa:** conceptualization, investigation, validation, resources, supervision. **Hidekazu Takahashi:** investigation, resources. **Masayuki Hiraki:** investigation, resources. **Yozo Suzuki:** resources, investigation. **Shinichi Yoshioka:** investigation, resources. **Mitsuyoshi Tei:** investigation, resources. **Shu Okamura:** investigation, resources. **Yukako Mokutani:** investigation, resources. **Atsushi Naito:** investigation, resources. **Shinji Tokuyama:** resources, investigation. **Takamichi Komori:** investigation, resources. **Akinobu Yasuyama:** resources, investigation. **Genta Sawada:** investigation, resources. **Yujiro Fujie:** investigation, resources. **Shinya Yamashita:** investigation, resources. **Naotsugu Haraguchi:** investigation, resources. **Yusuke Matsuura:** investigation, resources. **Chu Matsuda:** investigation, resources. **Yoshinao Chinen:** investigation, resources. **Tatsushi Shingai:** investigation, resources. **Katsuki Danno:** investigation, resources. **Nobuo Tanaka:** investigation, resources. **Kazuya Sakata:** resources, investigation. **Kohei Murata:** investigation, supervision. **Yuichiro Doki:** investigation, supervision. **Hidetoshi Eguchi:** investigation, supervision.

## Funding

The authors have nothing to report.

## Ethics Statement

This study was approved by the Ethics Committee of The University of Osaka (Approval No. 23323(T31)‐3). All institutions above granted the approval.

## Conflicts of Interest

Yuichiro Doki is an editorial board member of *Annals of Gastroenterological Surgery*. The other authors declare no conflicts of interest.

## Supporting information


**Table S1:** ags370226‐sup‐0001‐TableS1.docx.

**Table S2:** Comparison of *RAS* mutation status between MLH1‐proficient dMMR and MSS/MSI‐L/pMMR tumors.

**Table S3:** Comparison of Characteristics Between Tested and Untested Groups.
