# Supplementary material for: Prevalence of MSI‐H/dMMR Colorectal Cancer in Japan: Data From the Clinical Study Group of the University of Osaka‐Colorectal Registry
Source: Ann Gastroenterol Surg. 2026 May 24;10(5):1538–45. doi: 10.1002/ags3.70226 (PMC13394795; doi:10.1002/ags3.70226)
Supplement: Supplementary file 1 [file AGS3-10-1538-s001.docx]

| Supplementary table 1. | | | |
| --- | --- | --- | --- |
| MSI-H/dMMR | | | 137 |
|  | MSI-H | | 39 |
|  | dMMR | | 98 |
|  |  | MLH1 and PMS2 deficient | 72 |
|  |  | MLH1 and PMS2 and MSH6 deficient | 3 |
|  |  | MLH1 deficient | 2 |
|  |  | PMS2 deficient | 6 |
|  |  | PMS2 and MSH6 deficient | 2 |
|  |  | PMS2 and MSH2 deficient | 1 |
|  |  | MSH2 and MSH6 deficient | 8 |
|  |  | MSH2 deficient | 2 |
|  |  | MSH6 deficient | 2 |
|  | Abbreviations: dMMR, deficient mismatch repair; MSI, microsatellite instability | | |
