# Supplementary material for: Prevalence of MSI‐H/dMMR Colorectal Cancer in Japan: Data From the Clinical Study Group of the University of Osaka‐Colorectal Registry
Source: Ann Gastroenterol Surg. 2026 May 24:10.1002/ags3.70226. Online ahead of print. doi: 10.1002/ags3.70226 (PMC13394795; doi:10.1002/ags3.70226)
Supplement: Supplementary file 2 — Table S2: Comparison of RAS mutation status between MLH1‐proficient dMMR and MSS/MSI‐L/pMMR tumors. [file AGS3-9999-0-s001.docx]

| Supplementary Table 2. Comparison of *RAS* mutation status between MLH1-proficient dMMR and MSS/MSI-L/pMMR tumors | | | |
| --- | --- | --- | --- |
|  | **MLH1-proficient dMMR** | **MSS/MSI-L/pMMR** | **p value*** |
| *KRAS* mutant | 5 (23.8%) | 507 (38.2%) | 0.257 |
| Other *RAS* mutation | 0 (0%) | 49 (3.7%) | 0.618 |
| *RAS* wild-type | 6 (28.6%) | 636 (47.9%) | 0.083 |
| Unknown | 10 (47.6%) | 135 (10.2%) | <0.001 |
| Note: MLH1-proficient were defined as cases with intact MLH1 expression and loss of one or more other mismatch repair proteins (MSH2, MSH6, and/or PMS2), Abbreviations: dMMR, deficient mismatch repair; MSS, microsatellite stable; MSI-L,microsatellite instability-low; pMMR, proficient mismatch repair | | | |
