# Supplementary material for: Prevalence of MSI‐H/dMMR Colorectal Cancer in Japan: Data From the Clinical Study Group of the University of Osaka‐Colorectal Registry
Source: Ann Gastroenterol Surg. 2026 May 24:10.1002/ags3.70226. Online ahead of print. doi: 10.1002/ags3.70226 (PMC13394795; doi:10.1002/ags3.70226)
Supplement: Supplementary file 3 — Table S3: Comparison of Characteristics Between Tested and Untested Groups. [file AGS3-9999-0-s002.docx]

| Supplementary Table 3. Comparison of Characteristics Between Tested and Untested Groups | | | |  |
| --- | --- | --- | --- | --- |
|  | **MSI/MMR testing** | |  | |
|  | **Tested (n=1464)** | **Untested (n=732)** | p-value | |
| **SEX** |  |  | 0.041 | |
| **Male** | 819 (55.9%) | 375 (51.2%) |  | |
| **Female** | 645 (44.0%) | 357 (48.8%) |  | |
| **Age, median (range)** | 73 [26–97] | 75.0 [35–99] | <0.001 | |
| **Body mass index, median (range)** | 22.1 [12.2–39.5] | 22.1 [12.0–35.0] | 0.982 | |
| **Tumor location** |  |  |  | |
| **right-sided colon** | 531 (36.3%) | 274 (37.4%) | 0.653 | |
| **left-sided colon** | 598 (40.9%) | 303 (41.4%) |  | |
| **rectum** | 335 (22.9%) | 155 (21.2%) |  | |
| **Stage** |  |  | <0.001 | |
| **0–I** | 181 (12.4%) | 355 (48.5%) |  | |
| **II** | 521 (35.6%) | 207 (28.3%) |  | |
| **III** | 491 (33.5%) | 134 (18.3%) |  | |
| **IV** | 271 (18.5%) | 36 (4.9%) |  | |
| Note: Left-sided colon includes rectosigmoid colon, Abbreviations: MMR, mismatch repair;  MSI, microsatellite instability | | | | |
